# Physicians Hand Book

**Published:** 1887-01

**Authors:** 


THE PHYSICIANS' HAND BOOK FOR 1887, by Wm. Elmer, M. D., and A. D. Elmer, M. D., New York: W. A. Townsend, Publisher. Price, $1.50.

This is one of the neatest and most serviceable of the many books of the kind that are gotten out for the physician's pocket; substantially bound in leather, and neither too large nor too small for the breast pocket. It contains, in addition to an abundance of space for *eutries of all kinds*, such as Record of Practice, (weekly 32 names), Obstetric Record, Diagnostic Record, Cash Account, and General Memoranda; a special classification of disease, giving the symptomatology of each ; Poisons, and the treatment ; Emergencies, and their treatment ; Examinations of the Urine (diagnostic), list of incompatibles ; medicinal weights and measures ; formula for hypodermic medication ; doses for inhalation ; a list of remedial agents (which is a complete list of "Materia Medica and Therapeutics" within itself), and a lot of specimen "extemporaneous prescriptions." A thorough examination of this little work will convince one of the great value to any one actively engaged in practice.

